# Effect of Salvia Officinalis L. and Rosmarinus Officinalis L. leaves extracts on anxiety and neural activity

**DOI:** 10.6026/97320630015172

**Published:** 2019-03-15

**Authors:** Zineb Choukairi, Tahar Hazzaz, Mustapha Lkhider, Jose Manuel Ferrandez, Taoufiq Fechtali

**Affiliations:** 1 Laboratory of Biosciences, Functional, integrated and molecular exploration, School Of Sciences and Technology - Mohammedia, Hassan II University of Casablanca; 2 Laboratory Of "Inteligencia Ambiental", Polytechnic University of Cartagena, Spain

**Keywords:** Anxiety, Rosmarinus Officinalis L, Salvia Officinalis L, neural activity, GABA

## Abstract

Anxiety, the illness of our time, is one of the most prevalent and co-morbid psychiatric disorder that represents a significant socioeconomic burden. Conventional treatment is associated with a number of side effects and there is a need to develop new therapeutic strategies. Therefore, it is of interest to investigate the modulating effects of Salvia Officinalis L. and Rosmarinus Officinalis L. leaves extracts on anxiety using different behavioral tests, and on neural activity using the Multi-electrode array technique. Data shows the decrease of the time of the immobility associated with a significant increase in the time spent in the center of the open field arena in the treated animals compared to the controls. The number of buried marbles has also decreased in the treated animals in the marble-burying test. On the other hand results also show a decrease of the neural activity explained by a decrease of the number of spikes after 24,48 and 72 h following the addition of 12,5 µg/ml of the plant leaf extracts to the neural culture. However, there were no spikes after the administration of 25µg/ml of the plants extracts.

## Background

Anxiety is a state of alertness, of psychological and somatic tension in response to an unpleasant feeling or anxiety-provoking situations. It is distinguished from anxiety disorders, which are pathological behavioral states in which the subject cannot control his anxiety [1]. Anxiety disorders are among the most common psychiatric disorders in the general population, with lifetime prevalence ranging from 9.2% to 28.7 % in various countries [2]. Unlike non-pathological anxiety, anxiety disorders are usually more intense and persistent. They often occur in the absence of real danger and they are associated with a distress [3]. The pathophysiology of anxiety disorders is not really well known, however, brain imaging studies suggest hyperactivity in limbic regions, such as the amygdala and the insula during the treatment of emotional stimuli, as well as an aberrant functional connectivity between these structures and other inhibitory structures in the brain like the medial prefrontal cortex. Different system are implicated, specially, serotoninergic and GABAergic systems. The occurrence of an unforeseen event, (not necessarily stressful); acts on the GABA receptors and decreases the regulatory influence, revealing the excitatory effects of adrenaline or serotonin. This activates the limbic structures and induces a feeling of anxiety that stressors such as cortisol can accentuate [1]. There are two therapeutic approaches for the treatment of anxiety, the pharmacological treatment and the cognitive behavioral psychotherapy.

Selective serotonin reuptake inhibitors (SSRIs) and serotonin and noradrenaline reuptake inhibitors (SNRIs) were considered the pharmacological treatment of choice [4,[ 5]. However, these agents are not always effective against severe symptoms, do not work quickly and are often associated with side effects. For these reasons, benzodiazepines and other drugs have been used as alternative or even preferred pharmacotherapy for anxiety disorders, although there are concerns about benzodiazepine dependence [6].

Cognitive-behavioral psychotherapy leads to changes in behavior and in the way of thinking, which can reduce vulnerability to anxiety disorders and reduce the risk of relapse after stopping treatment. In addition, cognitive-behavioral psychotherapy promotes an active attitude toward treatment. For these reasons, its therapeutic effects are more likely to last longer than those of pharmacotherapy [3]. Numerous studies have reported the effectiveness of this therapy on anxiety disorders including posttraumatic stress disorder, obsessive-compulsive disorder, specific phobia [7], panic disorders and generalized anxiety disorders [3].

However, since anxiolytic drugs act on the entire brain, they can have deleterious effects on other brain functions. On the other hand, other therapeutic approaches are not suitable and not accessible to all. It is important to develop new therapeutic strategies. Some patients prefer herbal medicine [8]. Actually, the use of complementary and alternative medicine has become common in the general population [9]. This, in addition to the side effects of conventional drugs, can be explained by other factors, both social and economic. A good use of medicinal plants is therefore essential for the discovery of new molecules necessary for the development of future drugs, this is the case of Hypericum, which is a phyto-medicament characterized by its inhibitory properties on the reuptake of serotonin, norepinephrine and dopamine. It is also MAO inhibitor and has an effect on the secretion of melatonin and an action at the level of sigma receptors playing a role in the regulation of emotions [10].

In this study, two medicinal plants are tested for their eventual anxiolytic activity, Rosmarinus Officinalis L. and Salvia Officinalis L. both from the family of Lamiaceae growing spontaneously in many areas of morocco. Sage and Rosemary are widely used in morocco for their medicinal or culinary properties; they have attracted the interest of many researchers who reported their anti-bacterial [11], anti diabetic [12], anti depressant [13] and anti tumoral effects [14, 15]. For this purpose, we used three behavioral tests such as the open field, the marble burying test and the light/dark test for the evaluation of anxiety in sprague dawley rats. In the other hand, the plants extracts are assessed for their ability to modulate the neural activity, using the multi electrode array technique on primary cortical culture.

## Methodology

### Plant material:

Aerial parts of Rosmarinus Officinalis L. and Salvia Officinalis L. were collected from the region of Mohammedia, Morocco. The leaves were dried in shade at room temperature and crushed in the blender.

### Extraction:

The dried and powdered leaves of Salvia and Rosemary were used for extraction with methanol in the soxhlet apparatus. Then the obtained extracts were concentrated using a rota-evaporator in order to eliminate the solvent.

### Animals:

Adult male Sprague Dawley rats, weighing 180-250g were housed in the animal facility of the school of sciences and techniques, Mohammedia, in a cage with controlled room temperature (22±2 °c). Food and water were available ad libitum with 12h/12h dark/light cycle. All experiments were in accordance with international ethical standards. Every effort has been made to minimize the suffering of the animals and to reduce the number of the animals used to a minimum during the study.

### Treatment:

The animals were randomly divided into three groups (n = 6 for each group). The control group received vehicle (DMSO), while the treated groups received 50 mg/kg body weight of total extracts of the studied plants by an i.p injection 30 min before each behavioral test.

### The open field test:

The open field test is a classic test for observing the non pathological anxiety [16] as well as the exploratory and locomotion activity [17] of animals. The animal is placed in the center of the open field, which is a square arena (100cm x 100cm x 30 cm). The floor is subdivided into 25 identical squares (20 cm x 20cm). It is considered that the device comprises a central region (9 squares) and a distal region (16 squares). The center of the open field is illuminated by a lamp of 75 w, placed 1m above the arena. The behavior of the animals is observed for a period of 10 min. The time spent in the center, the time of immobility and the number of the crossed lines is measured. The device is thoroughly cleaned with 70% alcohol after the passage of each rat in order to remove any trace that may influence the behavior of the next animal.

### The marble burying test:

The marble burying test is a useful paradigm of anxiety and obsessive compulsive disorder [18]. In this study, we used an experimental protocol similar to that described by Gaikward et al. [19]. The rats were placed in separate plastic cages (21 cm x 38cm x 14 cm) containing 5cm depth sawdust litter. 18 clean glass marbles were arranged evenly on the bedding. After 3 min of exposure to the marbles, the rats were removed and the number of the buried balls was counted. A marble is considered as buried if its two thirds were covered with the sawdust.

### Light/dark box test:

The light/dark box test device is divided into two compartments, the first one is black and covered by a black lid (27cm x 18 cm x29cm), while the second one is white, illuminated and uncovered chamber called the white compartment (27cm x 27cm x 29cm). The two chambers were connected by a small opening allowing the free transition of the animal. During the test, the animal was placed in the center of the dark compartment; the time spent in each chamber and the number of transitions between them are observed and measured for a period of 10 min.

### Cell Culture:

The cortical neurons isolated from E18 rat embryos were grown in MEM (Earle's) 50% containing 25 % HBSS, 25% HS Dese and penicilin/streptomycin 1:100. The cells were maintained at 37°C and 5% CO2 .

### Stimulation and recording of the neural activity:

**Figure 1:**
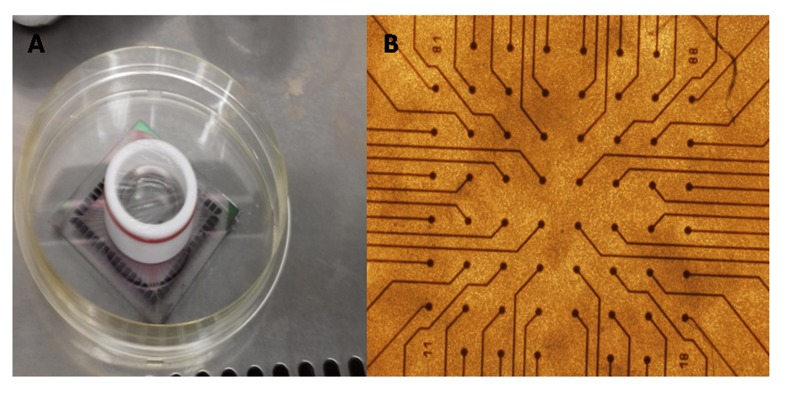
(A) Photo of the rat cortical neurons over multi electrode array; (B) Enlarged microscopic image

The neural stimulation and the activity recording were performed using the multi electrode array technique from Multi Channel Systems (MCS, Reutlingen, Germany). This technology comprises arrays of sixty 30 mm diameter electrodes and a 60 channel amplifier. The neural stimulation was performed using a dedicated two-channel stimulus generator capable to multiplex the signals which allows stimulating all the electrodes in almost a parallel process. The hardware is designed in such a way that the temperature is set at 37(°c), playing the role of amplifier, stimulator and temperature controller at the same time. MEA_Rack, MC_ Stimulus, MC_RACK software were used to carry out the different tasks, also provided by MCS. The spike sorting was performed using NESO-Neural sorter software, which make it easy to select and delete the artifacts, visualize, analyze the recording and save the data in different formats (Figure 1).

### Statistical analysis:

**Figure 2:**
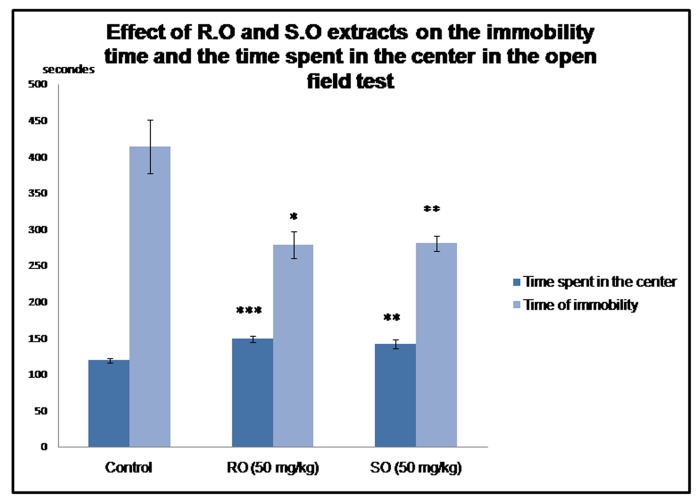
Effect of Rosmarinus officinalis L. and Salvia officinalis L. on the time spent in the center and the time of immobility in the open field test. Results are represented as mean ± SEM n = 6.

The statistical analysis was performed by specialized software SIGMA PLOT. Comparisons between the different groups were performed using the ANOVA followed by a post-hoc test. The degree of significance is represented on each graph by stars *** p <0.001 (highly significant); ** p <0.01 (very significant) * p <0.05 (significant)The open field test: Rosmarinus officinalis L. and Salvia Officinalis L. methanolic extracts reduce the time of immobility and increase the time spent in the center of the open field arena. The open field test was used to investigate the effect of R.O and S.O leaves extracts on anxiety and locomotor activity. The results show that the methanolic extracts of the studied plants significantly reduced the time of immobility comparing to the controls (H=8.246, p=0.016). The students t-test was used to analyze the difference between the groups separately, the results show a significant difference (p=0.013) between the controls and the R.O treated rats and a very significant difference between the controls and the S.O treated animals (p=0.01) (Figure 2). Furthermore, methanolic extracts of both salvia and rosemary have significantly increased the time spent in the center of the device (H=12.065, p=0.002). The comparison between the controls and the animals treated by rosemary resulted in a highly significant difference with p=<0.001, and a very significant difference with p=0.003 between the salvia group and the control group.

### The open field test: 

**Figure 3:**
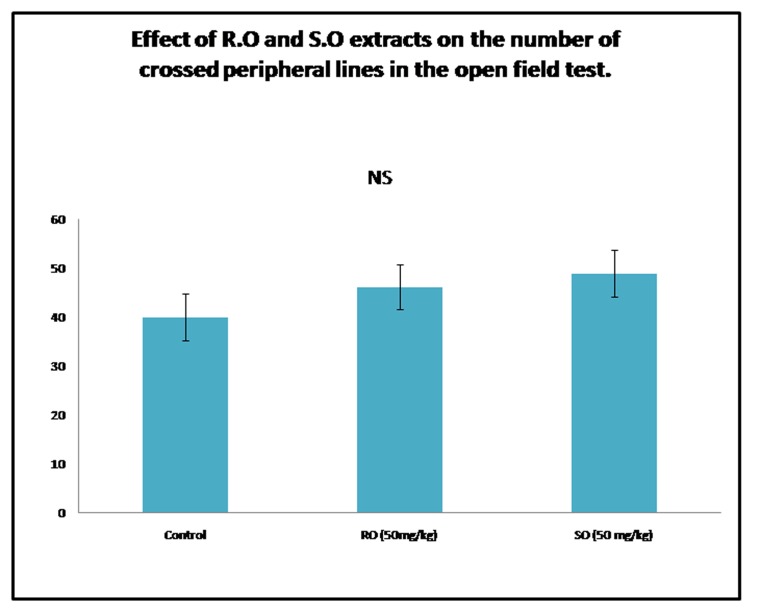
Effect of Rosemary and Sage leaves extracts on the number of crossed lines in the open field test. Results are represented as mean ± SEM n = 6.

R.O and S.O leaves extracts doesn't affect the number of the peripheral lines crossed in the open field. The number of the lines crossed in the open field test provides information about the locomotor activity. The effect of rosemary and sage on this parameter is represented in the following graph (Figure 3). The number of the peripheral lines crossed decreases in the control group compared to the treated groups. However, the statistical treatment reveals that the difference between the mean values of the groups is not important enough to reject the possibility that it is due to random variability. (H = 1.852, p = 0.396). Hence, the ip injection of the methanolic extracts of Salvia and Rosemary reduced the degree of anxiety without altering the loco motor activity of the rats.

### The marble burying test:

**Figure 4:**
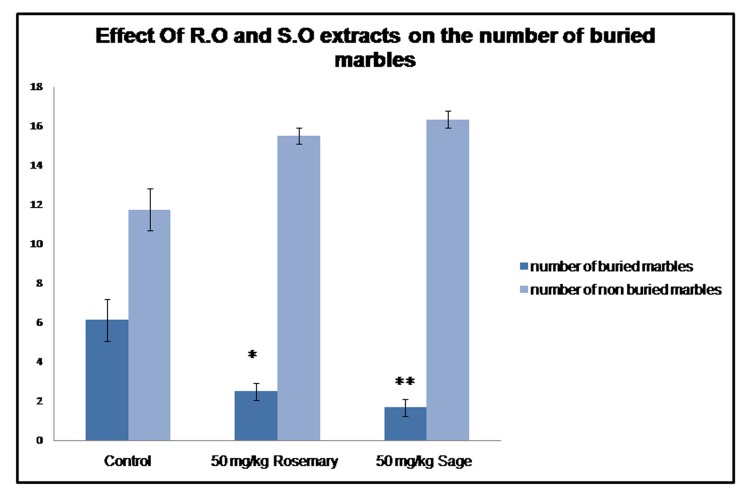
Effect of Rosemary and Sage leaves extracts on the number of buried marbles in the marble burying test.

 The ip injection of R.O and S.O leaves extracts reduced the number of buried marbles. The marble burying test provides information about anxiety and obsessivecompulsive disorder. In general, rodents tend to burry objects they perceive as potentially dangerous. Thus, when new objects are placed in their cage, the animals bury them with the litter, this behavior is attenuated by anxiolytic drugs, therefore, this psychological state is measured by the number of buried marbles, the more they are, the more the animal is anxious. In this study, the marble burying test was used as a tool to evaluate the effect of Salvia and Rosemary on anxiety. Our results represented in Figure 4 show that the number of buried marbles decrease in the plantstreated animals compared to the controls (H=9.999, p=0.007).

### The Light/dark box test:

 Salvia Offiicinalis L. and Rosmarinus Offiicinalis L. leaves extracts reduced the number of transitions between the two compartments and increased the time spent in the white one. The treatment of the rats with R.O and S.O leaves extracts increased the time spent in the center by the rats in the light chamber of the device and reduced the number of transitions between the compartments compared to the vehicle treated rats.

### Multielectrode array technique:

**Figure 5:**
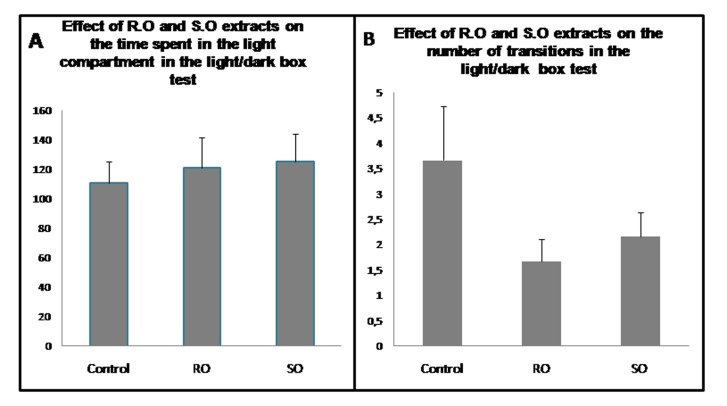
Light/dark box test. (A) Effect of the Rosemary and Sage leaves extracts on the time spent in the light compartment in the light/dark box test. (B) Effect of Salvia Officinalis L. and Rosmarinus Officinalis L. leaves extracts on the number of transitions in the light/dark box test.

**Figure 6:**
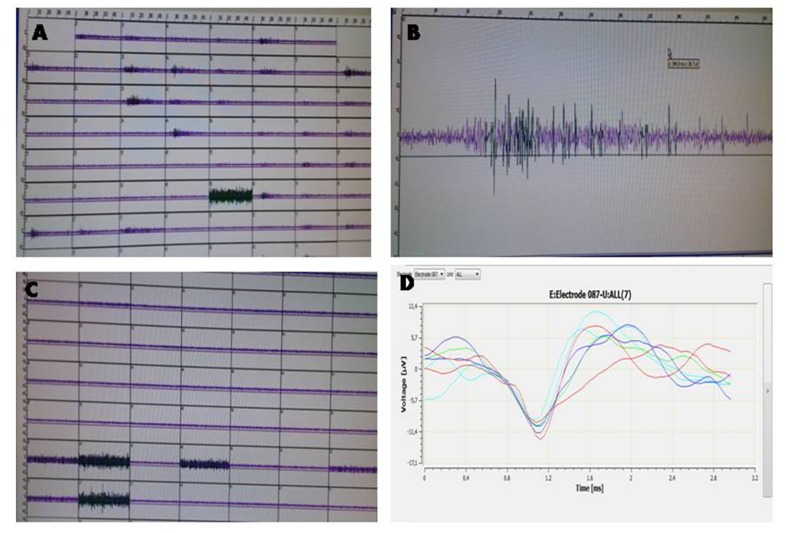
Recoding of a neuronal activity from cortical neurons (primary culture). (A) Cortical neuron contained in their medium (control). (B) Detailed image of a single site (control). (C) Cortical neuron contained in their medium after the addition of one the studied extract. (D) Example of a spike sorting from a precise electrode.

 Decrease of the neural activity following the administration of S.O and R.O leaves methanolic extracts. The multielectrode array technique was used in order to investigate the effect of R.O and S.O on neural activity. Our results show a decrease of the neural activity explained by a diminution of the number of spikes after 24, 48 and 72 h following the addition of 12.5 µg/ml of the plants extracts to the neural culture, however, we couldn't recorded any spikes after the administration of 25 µg/ml of the extracts to the medium (Figure 5, Figure 6).

## Results and Discussion

Sage and Rosemary have been extensively used either for their culinary or medicinal properties. Numerous studies have reported their biological effects but their central effects especially on psychological disorders are still controversial. It is of interest to investigate to investigate the modulating effect of Salvia Officinalis L. and Rosmarinus Officinalis L. Leaves extracts on anxiety together with their effect of neural activity. Our results suggest that both Sage and Rosemary exert an anxiolytic effect explained by the decrease of the immobility time and the increase of the time spent in the center of the open field maze, which is one of the most commonly used tests to measure different behavioral aspects in rodents. The results obtained in the marble burying and the light/dark test also reveal the anxiolytic effect of the studied extracts. On the other hand, we report a decrease in the neural activity following the administration of the R.O and S.O extracts to the primary neural culture medium, in a dose-dependent manner, using the multi electrode array technique. The cerebral cortex contains different types of neurons such as pyramidal cells having glutamate as the main excitatory neurotransmitter and Purkinje cells which are gabaergic neurons. Therefore, the decrease of the neural activity may be explained by the ability of certain compounds contained in the plant extracts to modulate the receptors of these neurotransmitters.

Indeed, it has been demonstrated that GABA receptors, are modulated by compounds isolated from Salvia Triloba L. (Ursolic acid, carnosol, oleanolic acid, salvigenin, rosmanol and hispidulin) [20]. On the other hand, Salvia Officinalis L. studies revealed that its constituents represent a strong affinity to benzodiazepine receptors in the human brain [21]. Abdelhalim and collaborators [22] have also reported that the main components of rosemary and sage like salvigenin, rosmanol and crisimaritine act as positive modulators of GABA-A receptors via three different sites. Moreover, these compounds were proven to have an anxiolytic effect, in a posterior study, using the elevated plus-maze and the dark/light box test [23]. Even if the neuroanatomical substrates involved in anxiety and its pathophysiology are very diverse and are still discussed, there is evidence that anxiety behavior is associated with hyperactivity in limbic region in the brain such as amygdala and insula [1]. Moreover, many studies have shown the role of GABA in modulating fear. Rodriguez Manzanares and his collaborators [24] have shown that GABAergic activity in the amygdala is attenuated by acute stress. The action mechanism of benzodiazepines, the most widely used pharmaceutical treatment of anxiety, consist of improving the GABAergic transmission by binding to the a and d subunits of the GABA-A receptors [25] which therefore increase the permiability of the nerve cells to chlorine resulting in a decrease in the nerve cell excitability [26].

## Conclusion

Taking together, it can be concluded that the results obtained in the various anxiety tests could be strongly related to the results obtained in electrophysiology. Thus, the decrease of neuronal activity could be at the origin of the anxiolytic effect observed and this through the improvement of the gabaergic transmission in certain regions of the brain.

## Conflict of Interest

Authors declare no conflict of interest
